# Asiatic Cholera

**Published:** 1847-10

**Authors:** 


*Asiatic Cholera.*—The Indian mail, just arrived, announces, in letters from Dinapore, that the cholera was ravaging her Majesty's 98th Regiment; 64 persons died in the month of May; there were 170 in the hospital daily. According to recent intelligence, published in the Gazette of Augsburg, the Asiatic cholera was still prevailing at Tiflis, where it has occasioned many deaths.—*Ibid.*

